# Chicago Veterinary Society

**Published:** 1898-03

**Authors:** 


### SOCIETY PROCEEDINGS.

### CHICAGO VETERINARY SOCIETY.

The regular monthly meeting was held on February 10, 1898. Meeting called to order by Dr. R. G. Walker, President, twenty-one members being present.

Under "Remarks by the President," Dr. Walker said that he was greatly surprised at the result of the vote cast as to the retention or expulsion of Drs. B. A. Pierce and W. E. McGraw, assistants to a non-graduate State Veterinarian; that had the vote been a tie the President would have most certainly voted for expulsion. That the retention of these members was antagonistic to all previous sentiments shown by the Society; that under new business a motion for reconsideration of the vote would be in order, the same to be voted upon at the next regular meeting if the vote for reconsideration was carried.

The President next presented a letter from the Secretary, Dr. James Henderson, resigning from the Society, as he intended leaving America to accept a desirable position in Scotland. It was moved and seconded that a committee of five members be appointed by the President to draft resolutions of thanks to Dr. Henderson for his efforts and work in the behalf of the Society, and regrets at his necessary resignation; carried.

The nominations for Secretary being in order, it was moved and seconded that Dr. Campbell be elected to the office, as his departure from Chicago had been postponed; voted and carried.

The committee appointed to draft the resolutions of thanks to Dr. Henderson were Drs. Hughes, Robertson, E. L. Quitman, Ryan, Baker.

There was no report from the Secretary or the Treasurer.

Regular programme, "A Guide to the Veterinarian in Examination for Soundness." The programme was opened by Dr. Worms on "Salivary Fistulæ, Salivary Calculi, Nasal Polypi, or Obstructions, Nasal Cysts, Nasal Catarrh, Partial Amputation of the Tongue, and Lolling and Paralysis of the Same." The paper was an extremely interesting one, and one to which the Doctor had evidently given great care and thought. The discussion was brisk and interesting, nearly all present taking part. Moved and seconded to close the discussion. Dr. Robertson next read his paper on "Osteoporosis, Enlarged Superior and Inferior Maxillary Bones, and Fractures Interfering with the Function of Same, Paralysis of the Lips, and Torn Commissure." The Doctor also mentioned facial expression and the general expression of the head, which would show the mental endowment of the animal as to vice, etc. A sharp discussion was occasioned by this excellent paper. On motion, the discussion was closed.

The next order of business being new business, it was moved by Dr. Nelson, seconded by Dr. Ryan, that a reconsideration of the vote taken at the last meeting in regard to the expulsion of Drs. Pierce and McGraw be had. A standing vote being taken, on count it was ascertained that eleven members desired reconsideration, while five did not. The vote being two-thirds affirmative, a new vote on expulsion will be held at the next regular monthly meeting. Owing to a spirited discussion as to members voting who had not paid their dues, it was ruled by the President that a delinquent member could not vote. Motion for appeal was taken to this ruling by Dr. E. L. Quitman, seconded by Dr. Dubia; voted and carried. The President stated that in making this ruling he was only living up to the by-laws of the Society as appears in Article IV, Sections 1 and 3, and that the appeal taken was not from the President, but from the Society's own by-laws; that unless these by-laws were lived up to and enforced by the members, he should tender his resignation. This was followed by a withdrawal of the motion by Dr. Quitman, with the hope that the seconder would also withdraw his second to the motion, which was not done. On motion, adjournment.

L. CAMPBELL, D.V.S.,
Vice-President and Secretary.

*Dr. James Robertson's Paper.*

OBSERVATIONS ON THE FACE IN EXAMINATION FOR SOUNDNESS. As the horse is gifted with the power to think, to reason about things and objects of his own sphere, to recognize facts of his experience and memory, it must be evident to every one that any examination as to soundness would be incomplete without a careful consideration of his mental endowments. These qualifications we may learn largely through a study of the expressions of the face. The parts most particularly charged with the manifestations of the internal state of the animal are the eyes and the eyelids, the ears, the nostrils, the lips, and the mouth. These organs, by the different attitudes they assume, deject by turns, gentleness, vivacity, anger, sadness, joy, pain, fear, courage, ferocity, indifference, and stupidity. The importance of the mental make-up of a horse becomes manifest when we consider the very close relationship existing between many of these mental conditions and pathological conditions that are influenced by them.

Excessive fear and ferocity, if chronic, are generally accompanied by disturbances of some or all of the vital, digestive, secretive, or excretive organs. Fright prepares the way for disease by undermining the nervous forces and weakening resistance, and the consequences of excessive anger or viciousness we all know result in the disturbance of every faculty and function. The action of the heart is seriously impaired, digestive processes are instantly checked, and do not proceed until the natural circulation of the blood is restored.

Where there were mental aberrations that, in my judment, would interfere with his health or usefulness, I would declare the horse unsound.

Osteoporosis, enlarged superior or inferior maxillary bones, and fractures interfering with function, I would consider unsound.

*Discussion of Dr. Robertson's Paper.*

Dr. E. L. Quitman: If Dr. Robertson takes into consideration the psychic condition and facial expression of animals, I would like to ask what guide he would have in examining mules.

Dr. Robertson: I am somewhat surprised that a veterinarian, who has to depend so largely on his knowledge of the animal from what he can see and find out of the pathology, should ask such a question. No veterinarian pretends to diagnose a case on the thermometer and pulse only.

There is a psychological condition that can be applied to animals—some say they have a soul; others that they have not. There is an expression in all animals by which, to a certain extent, the things that are passing through their minds can be learned, and I am surprised that a veterinarian should ask how this can be applied to a mule. It has been my misfortune not to be much associated with mules, and, therefore, I have not been able to study their physiognomy. When you consider the number of strange horses that are led into the ordinary blacksmith-shop, it is astounding that you never hear of a horseshoer being kicked. It is because the horseshoer knows pretty well how to behave himself around certain horses, and we all know the disposition of a horse the more we are associated with him. Veterinarians who are careless and rough seldom meet with success in the treatment of horses, on account of their actions. I know one who is careless and rough in the handling and treatment of horses, and I have seen horses become extremely nervous on account of it, and I contend that this psychological condition should be carefully observed. I do not pretend to be a physiognomist and to read expressions, but every horse has a general expression that leads us to be careful in going around him. I for instance, now recall a case where a horse will drive double under ordinary circumstances for fifteen or twenty miles, but if you hitch him into a top-buggy you cannot drive him over a mile. The reason is that, one day, when he was so hitched the top-buggy caught in a hook and he became frightened. Chronic kickers are of a very highly nervous temperament and are vicious, and I claim this is very essential in examining a horse for soundness. I would reject such a horse as unsound.

Dr. Quitman related the story of a pleasant mule which was very vicious when meddled with.

Dr. Hawley: Does Dr. Robertson mean to say that an animal can be psychologically unsound?

Dr. Robertson: Yes, sir. I consider that a horse can be psychologically unsound. The line is well drawn. A horse that is a dummy, and whose brain is not in proper working order, is unsound, and the line of demarcation is well marked. I have tried to show the connection between the mind and the work of the animal. I think the connection is very close. A man may be sound in body, but may have paresis. I think it necessary that a horse should have a sound mind and body, and we must take note of the horse's mind. Excessive viciousness I regard as an unsoundness. We must take anything into consideration that affects the physiological combination. A horse must have a sound body and mind. I have rejected several horses on that account.

*Dr. A. C. Worms' Paper.*

Partial Amputation of the Tongue. This condition is frequently met with, and is due to mechanical injuries. Moreover, stablemen, in order to control unruly or sensitive horses during cleaning, not infrequently pass a cord around the tongue. If this be sharply pulled, the tongue may easily be cut through, and the thinner the cord the more easily does the accident occur. Snaffle-bits produce the same effect, especially if worn. The tongue may also be injured by sharp or displaced teeth.

Rupture of the Frænum Linguæ sometimes occurs in horses, resulting in suppuration, abscess formation, and the production of fistulæ. Steffen, a foreign veterinarian, saw the point of a foal's tongue become gangrenous and slough after having been violently handled during some dental operation. His report of the case points to a bloodvessel having been ruptured.

The diagnosis presents no difficulty. The irritation in the mouth, salivation, want of appetite, or slow, cautious mastication readily indicate the nature of the injury and its extent. Healing is usually rapid and certain, though transverse wounds of the tongue may leave a deep depression. But even this is no great drawback, and is only worth notice, inasmuch as the animal wastes food in eating, and the tongue may be lacerated if forcibly handled during examination.

But a portion of the tongue may be torn away in the first instance or later, and if the frænum linguæ be involved mastication will be rendered difficult.

Loss of the Point of the Tongue. Attempts to cure protrusion of the tongue have shown that in horses the removal of three-quarters of an inch causes no inconvenience, but where more is lost the animals are unable to bring the food between the back teeth. At times they seek to effect this by holding the head high in the air, like chickens when drinking; but at best some food must be wasted, and mastication takes longer. Graf, a foreign writer, records that a horse which had lost the point of the tongue had severe swelling of the remainder, accompanied by salivation and inability to eat solid food. Only fluids and mashes could be taken. When the wound had cicatrized, the stump only extended about three-quarters of an inch beyond the first molar. In three weeks the horse could again eat ordinary food, but took three times as long as formerly to do so. Ludecke, another foreign writer, described a similar case in which the tongue was lost as far as the commencement of the frænum, but nevertheless the horse could eat as usual. I regard such a horse as unsound.

Paralysis of the Tongue (glossoplegia). Inflammatory processes may interfere with the movements of the tongue, but its paralysis depends on injury to the hypoglossal nerve, which supplies with motor-filaments the collective muscles of the tongue and most of those of the hyoid bone. Wounds, abscesses, or inflammatory processes may affect the nerve at some point of its course or at its origin on the inferior surface of the medulla, and thus produce glossoplegia.

Kater, a foreign writer, saw one-sided paralysis occur in a foal which, three months before, had been wounded in the throat with a knife. On the left side the muscles of the tongue had so completely disappeared that at the point the upper and lower coverings of the mucous membrane were in contact. The paralysis is also seen during severe infections, as of contagious pleuro-pneumonia of the horse. In central paralysis both nerves usually suffer, and, of course, both sides of the tongue, for the two hypoglossal nerves arise very close together. In the horse, paralysis of the tongue sometimes accompanies acute meningitis or hydrocephalus. But all double-sided paralysis is not necessarily central. Diplegia occurs in horses whose tongues have been roughly handled and where both nerves have been injured.

The symptoms of one-sided paralysis are displacement of the tongue and difficulty in mastication and deglutition. In double-sided paralysis both acts become nearly impossible, particularly the latter. The tongue generally hangs from the mouth. In protracted cases the muscles atrophy, though, of course, in single-sided paralysis only those of the paralyzed side suffer. The disease must not be confounded with the so-called "protrusion," where the tongue is voluntarily lolled out of the mouth. Paralysis is shown by distortion and inability to retract the tongue. The prognosis is unfavorable.

SALIVARY FISTULA. Wounds of the salivary glands and their ducts often fail to heal because the continual flow of saliva pushes aside the granulations and hinders closure. The gland-epithelium finally unites with that of the outer skin, and through the opening so formed saliva flows continually. Although the general condition of the animal is only slightly affected, much saliva escapes during eating, and mats the hair of the cheek, finally producing a blemish. This is an unsoundness.

SALIVARY CALCULI. These concretions form chiefly in the parotid, sublingual, and submaxillary ducts. They are caused by an accidental nucleus, such as a small piece of hay or corn, or other foreign body, penetrating the canal to which the salts of the saliva adhere, forming roundish or mulberry concretions, blocking up the duct, which becomes enlarged and distended with saliva. Sometimes an oat insinuates itself into the orifice of the parotid duct, producing distention of it by saliva, causing it to appear as a pendulous sac on the borders of the jaw. The concretion is only remarked after it has attained a considerable size. It appears as a hard, sharply-defined, slightly-movable swelling, generally lying on the outer surface of the jaw close to the front of the buccal opening of Stenon's duct, but sometimes on the posterior border of the under jaw. The salivary duct is usually distended behind the swelling, and when the flow of saliva is entirely shut off the gland is enlarged. Inflammation is seldom present, but may appear and lead to formation of abscesses. Unsound.

POLYPI. A polypus may be defined to be a tumor attached by means of a narrow pedicle, and the most familiar example is the nasal polypus attached to the superior part of the nostril. It is of soft consistence, bleeding when injured; often containing a limpid fluid in its centre, growing downward, filling the cavity of the nostril, causing much uneasiness to the animal and interfering very materially with the respiratory function. There is a discharge from the affected nostril, causing much uneasiness to the animal, often tinged with blood, especially during exercise or work. The animal makes a snuffling sound in its breathing, and frequently sneezes. The tumor cannot always be seen, but by growth becomes visible to the examiner. Sometimes it grows in the contrary direction and falls into the isthmus, and is apt to become temporarily lodged in the larynx, causing the animal to breathe with the greatest difficulty, with a loud, roaring sound, and often to fall down from exhaustion and want of breath. By great effort the animal coughs the obstructing tumor from the larynx into the fauces again, and then the roaring sound and difficulty of breathing disappear. This is an unsoundness.

NASAL CATARRH. Catarrh means a discharge of fluid from the mucous membrane. The form of catarrh under present consideration is at first a congestion, followed by inflammation of the mucous membrane of the nasal chamber—the Schneiderian or pituitary membrane, as it is specifically termed. The inflammation usually extends to the membranes of the sinuses of the head and often to the membrane of the larynx and pharynx, causing the complication of sore-throat. Quite frequently the membrane of the eyes is also affected, as evidenced by its congested condition and the flow of tears down over the cheeks; the nasal duct is lined with a continuation of the same membrane, and hence the inflammation of the eyes is only an extension of the disease over a continuous tract and not a special disease, as often supposed.

The membrane of the nasal duct being swollen, the effect of the inflammation or congestion, the tears cannot flow freely through it, therefore they escape from the eyes and flow over the cheeks.

*Symptoms.* The membrane at the beginning of the attack is dry, congested, and irritable; it is of a much deeper hue than natural, pinkish-red or red; soon a watery discharge from the nostril makes its appearance; the eyes, also, may be more or less affected, and tears flow over the cheeks. The animal may be dull, and frequently he emits a sort of sneezing snort, but does not cough unless the throat is affected. A few days after the attack begins the discharge from the nostrils changes from a watery to that of a thick mucilaginous state, of a yellowish white color, and may be more or less profuse. Often the appetite is lost, and the animal becomes debilitated. There is a rise in pulse and temperature. I regard such an animal unsound.

Nasal Cyst. This is a small globular tumor sometimes found within the nostril, under that part of the skin that is seen to puff or rise and fall when a horse is exerted and breathing hard. These tumors contain matter of a cheesy consistence, and are simple. If the tumor is well opened, and the matter squeezed out, nature will do the rest to perform a perfect cure. If the opening is made from the outside through the skin it should be at the most dependent part, but much the best way is to open the tumor from the inside. I regard such an animal as sound.

### *Discussion of Dr. Worms' Paper.*

Dr. Walker: I wish to understand whether we should drive a horse or not. I am interested in a driving-school on the West Side, and would like you to tell me to-night whether you think he should not be ridden in a saddle. I think in order to make a complete examination, you should not only drive a horse, but ride him.

Dr. Ryan: Do you consider side-pulling or tongue-lolling a disease?

Dr. Hawley: In bringing up the question of soundness or unsoundness, it must be viewed from two sides—the practical side and the theoretical side. Before going any further, I would like to ask this Association if it has decided on what is an unsoundness. I think it unprofitable to discuss it unless you define the term.

Dr. Walker: We have not arrived at that condition. It was considered that a committee would take that up further along, and then get the sentiments of the members here, and find out what they considered sound or unsound.

Dr. Hawley: In my opinion a horse may be technically unsound, but practically sound or serviceably sound. A bare spot is unsoundness—it is abnormal; a bad habit is unsoundness; kicking and balking, usoundness; a cribber, for all practical purposes, is sound—cribbing is a habit, not a disease. My definition is this: That any blemish or defect which injures the usefulness or selling-price is an unsoundness.

Dr. Worms said in his paper that if three-quarters of an inch of the tongue of a horse is removed, he will retain the food in his mouth. Technically that horse is unsound, because a part of his tongue is gone; practically he is sound. He can eat, and is in good condition, and would sell for just as much money as if he had the tongue. He says that tongue-lolling is not an unsoundness. It certainly is an unsoundness, not a disease. No man that cared for appearances would drive a tongue-loller through the street. Nasal cysts, he says, he would not consider an unsoundness; but in his definition he says they are abnormal. I would like to know if a nasal cyst is not an abnormality. It is an abscess, and contains pus, and if large enough would interfere with breathing.

Dr. E. L. Quitman: I would take exception to the remark that tongue-lolling is a habit. It is sometimes a symptom of decayed teeth. I have seen several cases where the decayed tooth was removed and the tongue would be carried normally.

Dr. P. Quitman: I wish to take an exception to the remark that tongue-lolling is a habit. It may be a disease—the horse cannot put his tongue back into the mouth. Such horses suffer a good deal with dry and cracked tongue. I have an idea it is a disease of the nerves or muscles that control the tongue, and I believe it is an unsoundness.

Dr. Walker: It is quite possible for tongue-lolling to be a habit, as there are tongue-lollers that can be stopped. I have seen tongue-lollers cured by putting two large pieces of leather under the tongue.

Dr. P. Quitman: That is curing by mechanical means. Some one interrupted and asked the difference between a habit and vice, to which Dr. Quitman replied that he understood that kicking was a vice in horses. A horse may be sound and be vicious; he is a kicker. Vice is not an unsoundness, according to the correct English definition of the word. Such a horse is dangerous and can be rejected, according to the law. Although you may claim that the horse is sound, according to law vice invalidates the sale.

Dr. Walker: English law is, "Sound, free from vice, and a worker."

Dr. Campbell: Will we accept or reject horses having the diseases mentioned if they occur?

Dr. Caspar: I differ with Dr. Worms somewhat in regard to the slight discharge from the nose in horses, as there are many horses coming from the country with a slight discharge from the nose, but are perfectly sound, and I would not reject them. Dr. Worms says anything which is not normal he would consider unsound. I do not consider it is fair to reject a horse with a temperature of 101.5° that has a slight discharge from the nostril. I see horses every day with a temperature of 102° F., with a slight discharge from the nose, and which are in every way sound, and I think it would be a great injustice to the seller to reject them.

Dr. Campbell: I would like to ask Dr. Caspar if he found a horse with a temperature of two degrees above normal, would he pass him?

Dr. Caspar: We do not take the temperature of animals out there. I still think it an injustice to reject a horse when he is perfectly sound and satisfactory in every respect, and satisfactory to the buyer, if he has a slight discharge from the nostril and a temperature of 102° F., and I would not reject him. My original argument was 101° or anything above normal, and I went to 101.5° or even 102°.

Dr. Worms: Dr. Caspar is discussing acute catarrh, where you get a temperature of 102°, mucous membrane of the eye injected; then comes the question whether it is acclimation fever or catarrh. In acute catarrh you do not get a purulent discharge. In chronic catarrh you get a purulent, thick, mucilaginous discharge, and it is an unsoundness, and I would not pass a horse with that discharge from the nose. A slight running at the nose, like you find in ephemeral fever and acute catarrh, I do not consider an unsoundness.

Dr. Quitman: The practical and technical division which Dr. Hawley made is a good one. For instance, I examined a high-priced horse some two or three months ago. He was extraordinarily sound, with the exception of a discharge from the nostrils and a temperature of 104°. The owner did not even go to the yards, but left the examination to me. After making same, I telephoned that the horse was sound, except for the time being he was sick, but thought he might recover. I told him of the danger of removal from the stable. He asked if I considered it safe for him to purchase. I told him it was safe if the animal was treated where he now was. In passing a horse for soundness it is well to put it to the common sense of the purchaser. A horse with ephemeral fever or acclimation fever is practically and technically unsound. This is so, for the slightest amount of work or exercise is apt to make him catch a fresh cold and bring on pneumonia or something else; at the same time, if the owner takes the chances, I would pass him. I drove the above horse with a temperature at 104°. I told the owner (the seller) he was sick, but would drive him at his risk; but, at the same time, you cannot give a dealer a fair show by passing a horse with fever at 104°.

Dr. Walker: You would not advise us to pass or drive a horse with a temperature of 104° *Ans.* No!

Dr. Walker: Why did you do it? *Ans.* We cannot always go according to law; I wanted to see if any lameness was present. The dealer told me to hitch him up and wind him.

Several made objections to winding a horse with a temperature of 104°.

Dr. Worms: I wish to add this remark about tongue-lolling: I consider a veterinarian is making a very careless examination if he does not drive the horse; of course, if a man is examining a truck-horse, it is a different thing. I never examine a driving-horse without riding behind him. I think a veterinarian is making an extremely careless examination if he does not drive the horse to discover whether he is a tongue-loller or side-puller, and, if either condition be present, he should tell the buyer and let him decide if he wanted the animal or not.

Dr. Quitman: It is very seldom, when called upon to examine a horse, that you will get to drive him; in fact, the party who buys him may drive him and not discover he is a tongue-loller. It is very hard to find whether a horse is a side-puller or tongue-loller unless you drive him. You may examine such a horse and say he is all right, if you do not drive him.

Dr. Hawley: Horses are sold at the stockyards every day with a temperature ranging from 101° to 104°. They are hitched up and winded time and again, and come out all right.

Dr. P. Quitman: Horse-dealers and veterinarians should look out for themselves. Some horses will retain their appetite, even though they be very sick. I would not pass a horse with a discharge from his nose, because it may subside within a few days or lead to something serious.

